# RNAi-based reverse genetics in the chelicerate model *Tetranychus urticae*: A comparative analysis of five methods for gene silencing

**DOI:** 10.1371/journal.pone.0180654

**Published:** 2017-07-12

**Authors:** Takeshi Suzuki, Maria Andreia Nunes, María Urizarna España, Hooman Hosseinzadeh Namin, Pengyu Jin, Nicolas Bensoussan, Vladimir Zhurov, Tawhid Rahman, Rebecca De Clercq, Pierre Hilson, Vojislava Grbic, Miodrag Grbic

**Affiliations:** 1 Department of Biology, The University of Western Ontario, London, Ontario, Canada; 2 Department of Plant Systems Biology, VIB, Ghent, Belgium; 3 Department of Plant Biotechnology and Bioinformatics, Ghent University, Ghent, Belgium; 4 Institut Jean-Pierre Bourgin, UMR1318 INRA-AgroParisTech, Saclay Plant Science, Versailles, France; 5 University of La Rioja, Logrono, Spain; Ecole Normale Superieure, FRANCE

## Abstract

RNA interference (RNAi) can be used for the protection against agricultural pests through the silencing of genes required for pest fitness. To assess the potential of RNAi approaches in the two-spotted spider mite, *Tetranychus urticae*, we compared 5 methods for the delivery of double-stranded RNA (dsRNA). These methods include mite feeding on either (i) leaves floating on a dsRNA solution, (ii) dsRNA-expressing plants, (iii) artificial diet supplemented with dsRNA, or (iv) dsRNA-coated leaves, and (v) mite soaking in a dsRNA solution. In all cases, the gene targeted for method validation was the *Vacuolar-type H*^*+*^*-ATPase* (*TuVATPase*), encoding a constitutively expressed ATP-driven proton pump located in the membrane. Down-regulation of *TuVATPase* increased mortality and/or reduced fecundity in all methods, but with variable efficiency. The most efficient methods for dsRNA delivery were direct soaking of mites in the dsRNA solution and mite feeding on dsRNA-coated leaves that mimics dsRNA application as a sprayable pesticide. Both resulted in a dark-body phenotype not observed in mites treated with a control dsRNA. Although with lower efficiency, dsRNA designed for *TuVATPase* silencing and expressed in transgenic *Arabidopsis* plants impacted the fitness of mites feeding on these plants. RNAi may thus be a valuable strategy to control spider mite populations, either as a sprayable pesticide or through transgenic crops. This comparative methodological study focusing on the induction of RNAi-based gene silencing in *T*. *urticae* paves the way for reverse genetics approaches in this model chelicerate system and prepares large-scale systematic RNAi screens as a first step towards the development of specific RNA-based pesticides. Such alternative molecules may help control spider mites that cause significant damages to crops and ornamental plant species, as well as other chelicerates detrimental to agriculture and health.

## Introduction

The chelicerates represent the second largest group of terrestrial animals after insects [1]. This basal lineage of the phylum Arthropoda includes horseshoe crabs, scorpions, spiders, mites and ticks that display a plethora of different lifestyles and include economically important species for human health and agriculture. Mite species exhibit a large range of adaptations including herbivory, predation, parasitism, detritivory and symbiosis [2,3]. With almost 50,000 species described by the end of 20^th^ century, and 0.5 to 1 million species estimated to exist, mites are one of the most diverse groups in the animal kingdom [4]. The two spotted spider mite (TSSM), *Tetranychus urticae* (Koch), is the first chelicerate whose complete genome was sequenced and annotated [5]. It is a compact genome of 90 Mbp (54% of which is protein coding sequence), with simple gene structure, complemented with several transcriptome databases. TSSM develops rapidly. It is easy to maintain in the laboratory and can be enriched in specific developmental stages. Because it is a major agricultural herbivorous pest, a strong research community supports the emergence of *T*. *urticae* as a versatile chelicerate model organism [6].

Most organisms whose genome has been sequenced in the last few years lack genetic tools available in established model systems (e.g. *Caenorhabditis elegans*, *Drosophila*, zebrafish and mouse). Thus, it is critical to develop high throughput platforms for reverse genetics, aimed at the functional characterization of unknown genes. Since its discovery, RNAi has been a useful tool to dissect gene function [7–9]. However, RNAi delivery is often a cumbersome process requiring the injection of dsRNA into the body of small animals to initiate gene silencing. In such cases, RNAi experiments remain limited to the silencing of individual genes, with the notable exception of a high throughput screen of over 5,000 genes in *Tribolium* [10]. Alternatively, streamlined dsRNA delivery methods resulted in relatively large assays. For example, a medium size screen (hundreds of genes) was performed in the coleopteran western corn rootworm *Diabrotica virgifera virgifera* based on an artificial diet laced with dsRNA [11,12]. Also, dsRNA delivery by soaking was implemented in a high throughput reverse genetic platform for *C*. *elegans* [13,14].

The genome of TSSM contains genes encoding for a complete RNAi processing machinery [5], including 2 Dicer homologs, pasha, Drosha and components of the RISC complex (including 7 orthologs of both *Argonaute* and *Piwi* genes). In contrast to other insects and similar to *C*. *elegans*, *T*. *urticae* possesses 5 copies of RNA-dependent RNA polymerase (RdRp) [5] required for the systemic spread of RNAi responses. It has already been shown that the maternal injection of either dsRNA or siRNAs in *T*. *urticae* induces RNAi in embryos and causes developmental aberrations consistent with the loss-of-function phenotype of the target gene [15]. RNAi is thus a valid reverse genetic approach in *T*. *urticae*. However, compared to other arthropods, for example *Tribolium* and *Oncopeltus* [16,17], it is difficult to inject dsRNA in TSSM females that are less than 0.5 mm in length. More recently, Kwon *et al*. [18] reported an alternative method for the oral administration of dsRNA via bean leaf discs floating on a dsRNA solution. Unfortunately, this protocol requires large quantities of dissolved dsRNA that must be replaced daily, making this method impractical for high throughput screens.

In the present study, we used the TSSM target gene *Vacuolar-type H*^*+*^*-ATPase* (*VATPase*), which encodes a protein that functions as a pump transferring protons across cellular membranes using the energy released by the ATP hydrolysis [19]. *TuVATPase* is a good target for methodological comparative analysis for several reasons. It has been previously used as a target for RNAi silencing in multiple arthropod systems, e.g. the Western corn rootworm (*Diabrotica virgifera virgifera*), the pea aphid (*Acyrthosiphon pisum*), the red flour beetle (*Tribolium castaneum*), the tobacco hornworm (*Manduca sexta*), the whitefly (*Bemisia tabaci*) and the Colorado potato beetle (*Leptinotarsa decemlineata*) [12,18,20–22]. These studies established the essential role of *VATPase*, demonstrating that effective silencing of *VATPase* expression leads to significant and measurable reduction of fitness across arthropod species. In addition, these studies showed that orally-delivered dsRNAs against *VATPase* induce RNAi. Finally, *TuVATPase* has already been shown to yield modest but significant mite mortality when silenced in *T*. *urticae* through the leaf floating method [18].

We describe several methods for dsRNA delivery into spider mites towards the development of reverse genetics platforms: soaking, which may be applicable to high-throughput screens; leaf coating, that mimics the application of dsRNA as a sprayable pesticide; and artificial diet for the administration of quantifiable amounts of dsRNA to TSSM. In addition, we tested whether transgenic plants expressing dsRNA may relay silencing into the mites.

We show that all 4 tested dsRNA delivery methods had greater efficiency in triggering RNAi in comparison to the leaf floating benchmark. The leaf coating and soaking methods, compatible with the high throughput experimental setups, had the greatest efficiency in silencing *TuVATPase*. Furthermore, because dsRNA expressed in transgenic plants or applied on the leaf surface induced RNAi affecting mite fitness, they may become valuable tools for crop protection against spider mites.

## Materials and methods

### *T*. *urticae* strains and rearing

Stock populations of *T*. *urticae* London reference strain and inbred lines were maintained on California red kidney beans (*Phaseolus vulgaris* L.) (Stokes, Thorold, ON) in a climate controlled chamber at L:D 16:8, 26°C, and 50% relative humidity (RH). Inbred lines were derived from isofemale lines that underwent 6 consecutive generations of mother-son mating.

### Preparation of developmentally synchronized mites

A detailed protocol for the synchronization of mite development (larval and adult emergence) has been described in an accompanied article [23]. Briefly, adult female mites were allowed to lay eggs for 24 h in a climate controlled chamber at L:D 16:8, 26°C and 50% RH. After 24 h, the adult females were removed and 1-day-old eggs were submerged in water. Three days later, water was removed and the 4-day-old eggs were allowed to dry. Newly-hatched larvae were collected after 3 h, yielding a tightly synchronized cohort. For the preparation of adults, adult female mites were placed onto a fresh detached bean leaf and were allowed to lay eggs at L:D 16:8, 26°C and 50% RH, for 24 h. Adult females were then removed from the leaf and the 1-day-old eggs were allowed to develop until female mites became teleiochrysalis (7–8 days). Teleiochrysalis were collected and incubated at 18°C and 100% RH for 24 h, after which they were returned to 26°C and 50% RH environment. Adult female mites, emerged within the 3 h upon transfer to 50% RH environment, were collected for experimentation.

### dsRNA fragments

**Fig 1:**
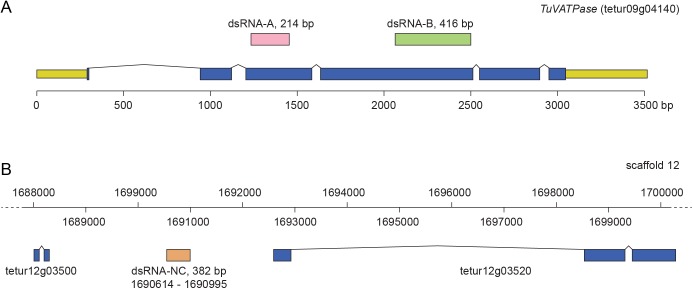
Fragments used for the synthesis of dsRNAs. (A) Schematic of the *TuVATPase* locus. DNA sequences used for the generation of dsRNA-*TuVATPase* are located in the 3^rd^ exon, fragment A (214bp), and in the 4^th^ exon, fragment B (416bp). UTR and coding sequences are shown in yellow and blue, respectively. (B) Schematic of the part of the scaffold 12 of *T*. *urticae* genome depicting the location of the 382 bp fragment that was used to synthesize dsRNA-NC.

We used 2 dsRNAs targeting the *TuVATPase* transcript to ensure the reproducibility and the specificity of the RNAi phenotype. Fragments corresponding to the coding region of *TuVATPase*, referred to as fragment A (an upstream fragment of 214 bp within the 3^rd^ exon of the *TuVATPase* locus) and fragment B (a 416 bp fragment within the 4^th^ exon) were chosen as templates for the preparation of dsRNAs (Fig 1). Furthermore, the fragment B corresponds to the dsRNA sequence previously used by Kwon *et al*. [18] enabling direct comparison of method efficiency between the previous report and this study. In addition, a 382 bp intergenic fragment spanning the region 1690614–1690995 of the genomic scaffold 12 [5] (Fig 1), was chosen as the template for the preparation of a negative control dsRNA, referred to as NC. A BLAST search against the *T*. *urticae* genome and transcriptome databases confirmed that the 382 bp intergenic sequence is unique and not transcribed [24,25].

### dsRNA preparation

The nucleotide sequences of *TuVATPase* (tetur09g04140), and intragenic region (negative control (NC), genomic coordinates: scaffold 12, position 1690614–1690995) were obtained from the ORCAE database [24]. Total RNA was extracted from the frozen mite females with the RNeasy Mini Kit (Qiagen, Valencia, CA) and cDNA was synthesized from 3 μg of the extracted total RNA with the SuperScript II cDNA Synthesis Kit (Thermo Fisher Scientific, Waltham, MA) and stored at −20°C. Fragments (A and B) of *TuVATPase* and a control sequence (NC) targeting an intergenic region (Fig 1) were PCR amplified with primers shown in Table 1, using cDNA and genomic DNA as templates, respectively, with Phusion DNA polymerase (NEB, New England Biolabs, UK). Amplified DNA fragments were purified with the Gel/PCR DNA Fragments Extraction Kit (Geneaid Biotech, New Taipei, Taiwan). Purified fragments were cloned in pLITMUS 38i vector (NEB, New England Biolabs, UK) containing T7 polymerase promotor sequences flanking multiple cloning sites to streamline dsRNA production. Inserts selected for the synthesis of dsRNA were re-sequenced to confirm their identity. RNA fragments were synthesized using 1 μg of DNA template with the TranscriptAid T7 High Yield Transcription Kit (Thermo Fisher Scientific) in 1.5 mL centrifuge tubes, denaturated at 95°C for 5 min, followed by slow cool-down to room temperature to facilitate formation of dsRNA. dsRNA was purified by phenol-chloroform extraction followed by ethanol precipitation [26]. dsRNA was dissolved in nuclease free water and quantified using NanoDrop (Thermo Fisher Scientific, Waltham, MA).

**Table 1:** Primers used in this study.

Cloning primers		
Primer names	Oligonucleotide sequence (5’ to 3’)	Fragment names (bp)
Tetur-VATP-F-A	CAGTTCTCCGAACCGGTAAA	A (214)
Tetur-VATP-R-A	CCACCGGTAATGTGACTACCA	
Tetur-VATP-F-B	CCGTGATATGGGTTACCATG	B (416)
Tetur-VATP-R-B	GAAGAGGTACGAAATCTGGG	
Tetur-sc12-F	GCCCTCTCCTGGTTGTAAACTT	Control, NC (382)
Tetur-sc12-R	CGACCCCATCAGGCTATTGA	
Spider mite RT-qPCR assay primers		
Primer names	Oligonucleotide sequence (5’ to 3’)	Primer efficiency
RP49 (tetur18g03590) F	CTTCAAGCGGCATCAGAGC	97.6%
RP49 (tetur18g03590) R	CGCATCTGACCCTTGAACTTC	
VATPase qPCR F	GGGTACCATCACATTCCTCG	104.1%
VATPase qPCR R	AATCGGTCTGGTTTGACGAAC	
Transgenic plant RT-qPCR assay primers		
Primer names	Oligonucleotide sequence (5’ to 3’)	Primer efficiency
PEX4 (AT5G25760) F	GCTCTTATCAAAGGACCTTCGG	99.2%
PEX4 (AT5G25760) R	CGAACTTGAGGAGGTTGCAAAG	
VATPase qPCR F	GGGTACCATCACATTCCTCG	104.1%
VATPase qPCR R	AATCGGTCTGGTTTGACGAAC	

### Delivery of dsRNA

#### Leaf floating method

Delivery of dsRNA into mites was performed as described by Kwon *et al*. [18] with minor modifications. Solutions of dsRNA for the *TuVATPase* A, B and control sequences were prepared at the concentration of 160 ng/μL. Four hundred microliters of dsRNA solution was added in each well of a 24-well polystyrene plate (Corning, NY) and bean leaf discs (10-mm diameter) were floated on its surface. Newly-hatched larvae or newly-molted adult females were inoculated onto the floated discs (5 larvae/disc or 1 adult/disc). After inoculation, the plate was covered with a lid and maintained at L:D 16:8 and 26°C. Survival and development of the larvae and survival and fecundity of adults were recorded over 8 and 5 days, respectively. For RT-qPCR analysis, the adult females were collected into a 1.5-mL tube (at least 30 adults/tube) after 3 days of treatment. The collected samples were frozen in liquid nitrogen and stored at −80°C until RNA extraction. The biological assays on larvae and adults were conducted on 5 independent experimental runs, and the RT-qPCR analysis on 3 independent experimental runs. No leaf withering was observed after 8 days of floating on the dsRNA solution.

#### Leaf coating method

Six microliters of dsRNA solution (160 ng/μL), transcribed from the *TuVATPase* A, B or control sequence, were evenly distributed onto the upper surface of bean leaf discs (10-mm diameter). The surface of dsRNA-coated leaf discs was allowed to dry and were subsequently placed on water-soaked cotton in a polystyrene cup (94 mm in diameter, 57 mm in depth) incubated at 26°C, L:D 16:8 and 50% RH. Newly-hatched larvae (5 larvae/disc) or newly-molted adult females (1 adult/disc) were inoculated onto the dsRNA-coated discs. Survival and development of the larvae, and the survival and fecundity of the adults were observed for 10 days. For RT-qPCR analysis, the adult females were collected into a 1.5 mL tube at day 5 after transfer to dsRNA-coated leaf discs. The collected mites were frozen in liquid nitrogen and stored at −80°C until RNA extraction. The biological assays and the material collection for RT-qPCR analysis were conducted in 3 independent experimental runs.

#### Transgenic plants expressing a hairpin dsRNA

With the aim to induce RNAi through mite feeding on transgenic plants expressing a hairpin dsRNA, the fragment A (Fig 1), of the *TuVATPase* transgene was cloned as inverted copies into the pAGRIKOLA binary vector carrying the *bar* gene coding for BASTA resistance, and transformed into *Arabidopsis thaliana* plants [27]. Wild-type Kondara plants were transformed via the *Agrobacterium* floral dip method [28]. Seeds collected from the infiltrated plants were germinated and grown under a short-day photoperiod (L:D 10:14) with a photosynthetic photon flux density of 100–150 μmol m^−2^ s^−1^ at 24°C and 55% RH and the transformed seedlings were selected by daily spraying the BASTA herbicide (10 mg/L). Six independent T1 seedlings were self-pollinated. The collected T2 progenies were analyzed for the BASTA resistance to discriminate lines that segregate BASTA resistance as single or multiple genetic loci. Representative lines that segregated BASTA resistance as single (line 2–3) and multiple (line 2–1) genetic traits were used in further experiments. The progeny seedlings of the T2 2–3 line were subjected to BASTA treatment to select homozygous seed stocks with 100% resistant phenotype. The progeny of the T2 2–1 line was selected for BASTA resistance prior to experiments, to ensure the presence of a transgene in each experimental plant.

Detached youngest adult fully-expanded leaves were collected from 3-week-old transgenic and control (untransformed) *Arabidopsis* plants, and were infested with either 10 newly-hatched larvae (for determination of the developmental rate and mortality; total of 70 larvae/experimental run) or with one mated newly-emerged adult female mite (for determination of fecundity over 10-day period; total of 10 females/experimental run). These experiments were conducted in 3 independent experimental repeats. Detached leaves were replaced every other day. We used a London mite population adapted to *Arabidopsis* for these experiments, in order to enhance mite feeding.

For RT-qPCR analysis of *TuVATPase* transcript level, detached *Arabidopsis* leaves collected from either transgenic or control plants were infested with about 250 newly-hatched larvae. About 150 adult spider mites (collected in 3 independent batches of approximately 50) were recovered from a detached leaf setup with an aspirator after 2 weeks. Mites were flash frozen in liquid nitrogen and stored in −80°C until RNA extraction.

#### Artificial diet

The artificial diet was formulated as previously described [23,29,30]. Parafilm® M hemispheres were assembled with a custom built vacuum device as described in Suzuki *et al*. [23] and Jonckheere *et al*. [30], and 40 μL of diet was added in each hemisphere before sealing with Scotch tape. Individual hemispheres were placed in wells of the 24-well culture plate prefilled with 1 mL of water to create a water barrier preventing mite escapes. Diet was supplemented with 160 ng/μL of the dsRNA fragments (*TuVATPase* fragment A, B or control). Newly-hatched larvae or newly-emerged female adult mites were placed on individual diet-filled Parafilm® M hemisphere, and incubated at L:D 16:8, 26°C and 50% RH. Larval and adult survivorship was observed over 5 days in 3 independent experimental runs.

For RT-qPCR analysis, 100 newly-emerged female adult mites were fed on 4 Parafilm® M hemispheres filled with the artificial diet for 5 days. Mites were collected and placed into a 1.5-mL tube for subsequent RNA extraction. The collection was performed in 3 independent experimental runs.

#### Soaking

Twenty five newly-hatched larvae or 50 newly-emerged adult females were soaked in 25 μL and 50 μL of dsRNA solution (160 ng/μL; 0.1% v/v Tween 20) respectively, and incubated at 20°C for 2 h (larvae) or 24 h (adults) according to Suzuki *et al*. [23]. After soaking, mites were transferred onto bean leaf discs (10-mm diameter; 5 larvae/disc or 1 adult/disc) placed on water-soaked cotton in the cup with a polyethylene lid with 4 venting holes each covered with a gas-permeable filter (0.45 micron pore size; Milliseal; EMD Millipore, Billerica, MA), and incubated at L:D 16:8, 26°C and 50% RH. Survival of larvae and adults, larval development and fecundity of the adult females were recorded over 10 days. The biological assays were conducted in 3 independent experimental runs.

For RT-qPCR analysis, the adult females were collected into a 1.5-mL tube (at least 30 adults/tube) at 5 days after 24-h soaking. The collected samples were frozen in liquid nitrogen and stored at −80°C until RNA extraction. The collection and the RT-qPCR analysis were conducted in 3 independent experimental runs.

### Analysis of RNAi efficiency in inbred lines

Newly-emerged adult females from eleven inbred lines generated from the reference London population were soaked in a 1.5-mL tube (60–80 adults/tube) with 50 **μ**L of dsRNA solution (160 ng/**μ**L; 0.1% v/v Tween 20). Adult females soaked in the dsRNA solution were incubated at 20°C in a water bath for 24 h. After soaking, mites were washed in 100 **μ**L of double distilled water and transferred onto a bean leaf that was placed on top of water-soaked cotton in a cup with vented lid, and incubated at 26°C, L:D 16:8 and 50% RH. After 5 days, mites with dark and normal body color were evaluated for mortality; surviving mites from each phenotypic group were separated and placed on new fresh bean leaves. After 3 additional days, the fecundity from each group was evaluated. Each experimental run was performed with 4 replicates and the reference London population was used as a positive control.

### RT-qPCR analysis

Plant total RNA was extracted from approximately 100 mg of leaf tissue harvested from 3-week-old transgenic and control *Arabidopsis* plants, with the RNeasy Plant Mini Kit, including a DNase treatment (Qiagen). To assess the stability of transgene expression, RNA samples were collected from 3 leaves at 1, 4 and 7 days after they were detached from the original plant.

Mite total RNA was extracted with the RNeasy Mini Kit, including a DNase treatment (Qiagen). Two micrograms of total RNA was reverse transcribed with the Maxima First Strand cDNA Synthesis Kit for RT-qPCR (Thermo Fisher Scientific).

qPCR reactions were performed in 3 technical replicates for each sample with the Maxima SYBR Green/ROX qPCR Master Mix (Thermo Fisher Scientific). The RT-qPCR was performed on an Agilent Mx3005P qPCR instrument (Agilent Technologies, Santa Clara, CA). The reference plant gene was *PEX4* (AT5G25760), coding for a ubiquitin conjugating enzyme [31], while *RP49* (tetur18g03590), a ribosomal protein reference gene [32] was the reference gene for mite samples. Primer sequences and amplification efficiencies (E) are listed in Table 1. Cycle Threshold (Ct) values from 3 technical replicates were averaged to calculate the Ct value of each independent experimental run [33]. For plotting, expression value for each target gene (T) was normalized to the reference gene (R) and normalized relative quantity (NRQ) was calculated as follows: NRQ = (1+E_R_)^CtR^/(1+E_T_)^CtT^. NRQ values were then normalized to a mean of those in the control. Differences in the mean of normalized NRQ values between the control and treatments were analyzed with the Dunnett’s test (function glht, R package multcomp) in the R 3.2.5 software (R Core Team 2016).

### Data analysis of survival and life history parameters

Survival curves were calculated with the Kaplan–Meier method (function survfit, R package survival) with comparisons performed based on the log-rank test (function survdiff, R package survival). Results for the developmental times and fecundity are displayed as box-plots where central lines (second quartile, Q2) indicate the median of data, the distance between the box bottom (first quartile, Q1) and top (third quartile, Q3) indicate interquartile ranges (IQRs), and the whisker bottom and top indicate the minimum and maximum of data (except outliers that are outside the range between the lower (Q1−1.5×IQR) and upper limits (Q3+1.5×IQR) that are plotted as a white circle). Significant differences in the median developmental time and the median number of eggs laid between the control and other treatments were analyzed with the Wilcoxon–Mann–Whitney test (function wilcox.exact, R package exactRankTests). A significant difference in the proportion of color phenotype (normal or dark-body) was analyzed with the Fisher’s exact test (function fisher.test). For multiple comparisons based on the paired tests, the level of significance (*α*) was adjusted with the Bonferroni correction (*α*/*k*, where *k* is the number of pairs in the multiple comparison). Analysis was performed with the R 3.2.5 software (R Core Team 2016).

### Data analysis of *TuVATPase* RNAi effect on inbred lines

RNAi was induced by soaking newly-emerged adult females as described in the “Soaking” section. Number of alive and dead individuals, and phenotype (normal or dark-body) was assessed 5 days post soaking. Fecundity of surviving females was assessed over 3 days (days 5–8 after soaking). To build the heatmap, dead/alive and normal/dark-body counts were converted to proportion of total mites recovered after soaking procedure and hierarchical clustering analysis was performed based on Euclidean distance and with the average clustering method [34]. Fecundity data were scaled to the 0–1 range and the heatmap was organized according to survival and phenotype clusters. The Wilcoxon–Mann–Whitney test was applied to analyze the differences in mortality and normalized fecundity between phenotype and treatment classes. *P*-values were adjusted for multiple testing with Bonferroni correction and *k* = 9. Analysis was performed with the R 3.2.5 software (R Core Team 2016).

## Results

### Induction of RNAi in the leaf floating setup

**Fig 2:**
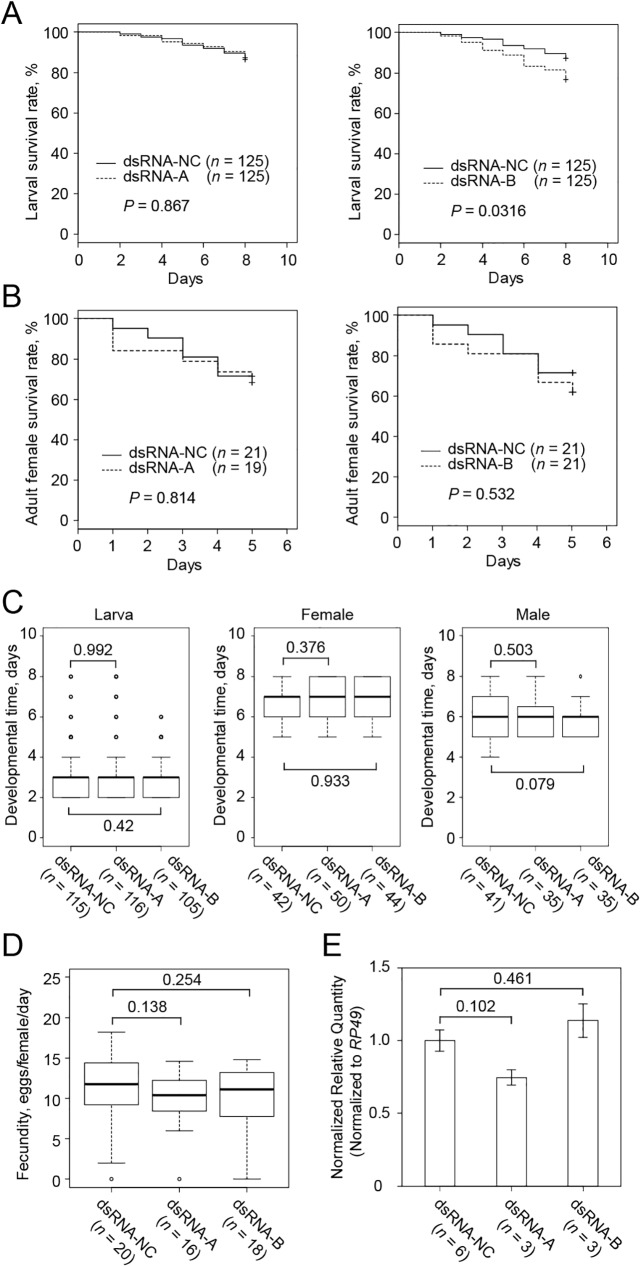
The effect of dsRNA-*TuVATPase* delivered by the leaf floating method. Larval (A) and adult female (B) survivorship when feeding on bean leaf discs floating on the solution of dsRNA-*TuVATPase-*A or dsRNA-*TuVATPase-*B (dashed lines), or dsRNA-NC (solid line). Data were collected from 5 independent experimental runs. Survival curves were plotted using the Kaplan–Meier method and compared using the log-rank test with Bonferroni correction (no asterisks, *P* > 0.05/2). (C) Developmental times of the larval (left) and adult female (middle) and male (right) mites and (D) mite fecundity. Data were collected from 5 independent experimental runs and were compared using the Wilcoxon–Mann–Whitney test with Bonferroni correction (no asterisks, *P* > 0.05/2). (E) *TuVATPase* gene expression relative to the expression of *RP49* reference gene. For the analysis of the gene expression, data were represented as mean±SE and analysed using the Dunnett’s test (no asterisks, corrected *P* > 0.05).

Delivery of dsRNA to mites through floating leaf discs is a current benchmark for RNAi in *T*. *urticae* [18]. This method and dsRNA directed against the *TuVATPase*-B sequence thus served as our reference baseline in this comparative analysis of alternative dsRNA delivery methods. The effects of dsRNAs were assessed by following the expression of the target gene together with indicators of mite performance: larval and adult survivorship, larval and adult developmental timing, and fecundity (Fig 2). As over half of the adults escaped from the floated discs and drowned in the surrounding solution at the end of the treatment (>6 days), the survival curves and fecundity measurements were limited to the first 5 days, only including mites that continuously fed on the floating discs during this period.

Although some of the measured traits tended to decrease, no significant difference was observed in the larval or adult performance parameters when *T*. *urticae* fed on leaf discs floating on the solution containing dsRNA-*TuVATPase*-A or dsRNA-*TuVATPase*-B, relative to the dsRNA-NC control, (Fig 2A–2D). Furthermore, no significant effects of dsRNA-*TuVATPase*-A or dsRNA-*TuVATPase*-B were observed on the relative level of *TuVATPase* expression (Fig 2E). Thus, the effects of dsRNAs homologous to the *TuVATPase* transcripts and supplied to the mites through floating leaf discs were non-significant and in accordance with the previously published data [18].

### Induction of RNAi through the artificial diet

**Fig 3:**
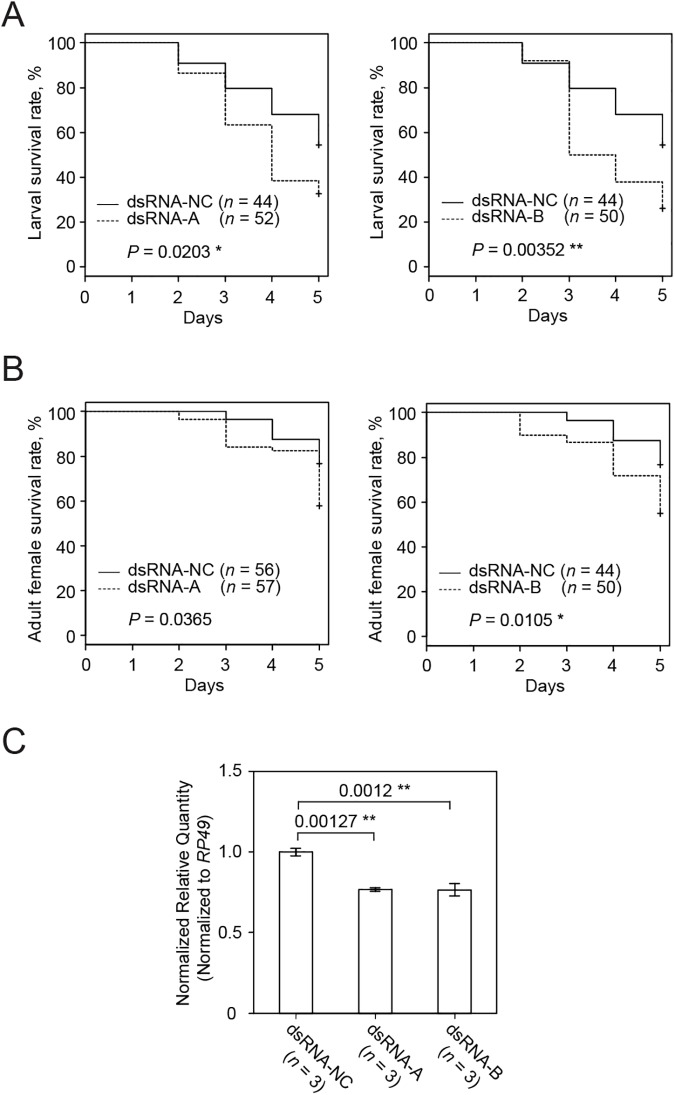
The effect of dsRNA-*TuVATPase* delivered through an artificial diet on mite survivorship and the endogenous *TuVATPase* gene expression. Larval (A) and adult female (B) survivorship during a 5-day feeding on artificial diet supplemented with dsRNA-*TuVATPase-*A (dashed line), dsRNA-*TuVATPase-*B (dashed line) or dsRNA-NC (solid line). Survival curves were plotted using the Kaplan–Meier method and compared using the log-rank test with Bonferroni correction (no asterisk, *P* > 0.05/2; *, *P* < 0.05/2; **, *P* < 0.01/2). (C) *TuVATPase* gene expression relative to the expression of *RP49* reference gene. For the analysis of the gene expression, data were represented as mean±SE and analysed using the Dunnett’s test (**, corrected *P* < 0.01).

Feeding of spider mite larvae and adults on the artificial diet supplemented with 160 ng/**μ**L of dsRNA-*TuVATPase*-B for 5 days resulted in significantly higher mortality of mites at both developmental stages compared to the dsRNA-NC control (Fig 3A and 3B). On the other hand, the application of dsRNA-*TuVATPase*-A resulted in the significant increase of larval but not of adult mortality (Fig 3A). Ultimately, the dsRNA-*TuVATPase*-A or -B reduced larval survivorship down to 30% after a 5-day feeding. The effects of dsRNA-*TuVATPase* on mite mortality correlated with the significant reduction of *TuVATPase* transcript level in these mites, down to 75% relative to mites fed on the artificial diet supplemented with the dsRNA-NC control (Fig 3C).

### Plant transgene induced RNAi

**Fig 4:**
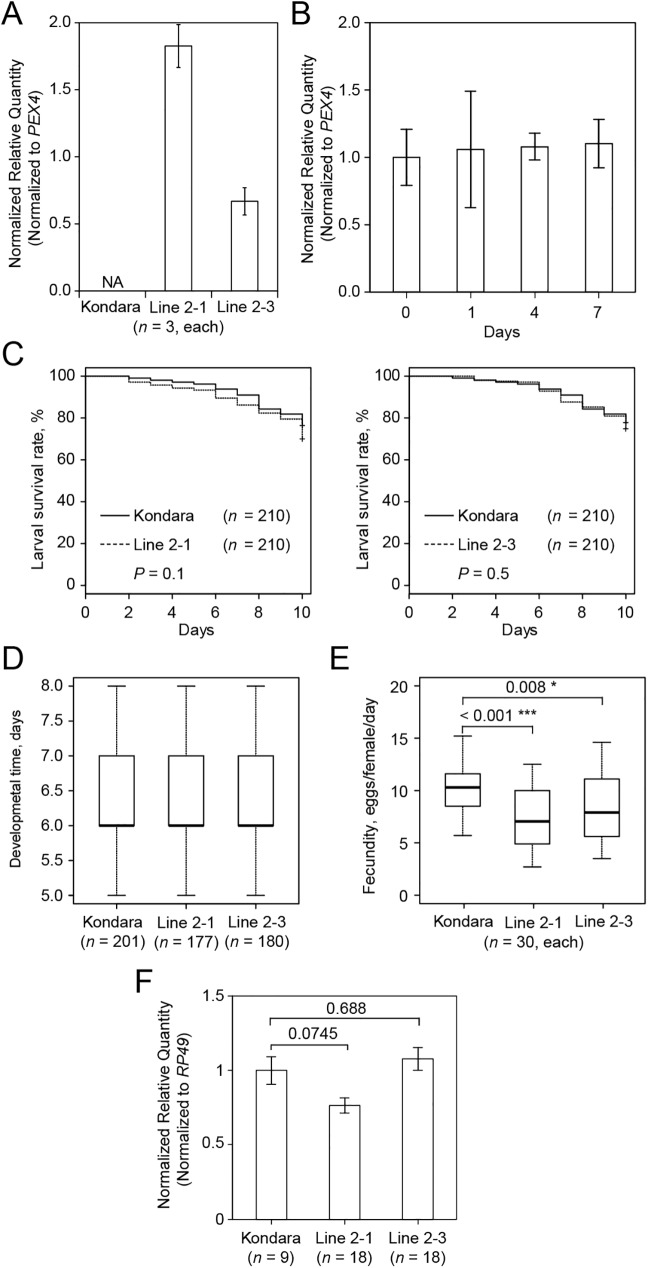
Mite performance and the endogenous *TuVATPase* gene expression upon mite feeding on transgenic plants expressing dsRNA-*TuVATPase* transgene. (A) Levels of dsRNA-*TuVATPase* transgene expression in 2 independent transgenic lines of *Arabidopsis* (accession Kondara), line 2–1 (carrying multiple transgene insertions) and line 2–3 (carrying a single transgene locus), relative to the expression of *PEX4* reference gene. Samples for the analysis of the gene expression were collected from 3 independent experimental runs. (B) Stability of dsRNA-*TuVATPase* transcripts in detached leaves of transgenic *Arabidopsis* plants (line 2–3) over 7 days. Levels of transgene expression were assessed by RT-qPCR and presented relative to the expression of *PEX4* reference gene. Samples for the analysis of the gene expression were collected from 3 independent experimental runs and data were analysed using the Dunnett’s test (no asterisk, *P* > 0.05). (C) Larval survivorship over a 10-day period upon mite development on detached leaves of control (Kondara; solid line) and transgenic *Arabidopsis* lines expressing dsRNA-*TuVATPase* transgene (dashed line). Data were collected from 3 independent experimental runs. Survival curves were plotted using the Kaplan–Meier method and compared using the log-rank test with Bonferroni correction (no asterisk, *P* > 0.05/2). (D) Developmental time of adulthood in mites feeding on detached leaves of control (Kondara) and transgenic *Arabidopsis* lines expressing dsRNA-*TuVATPase* transgene. Data were collected from 3 independent experimental runs and data were analysed using the Wilcoxon–Mann–Whitney test with Bonferroni correction (no asterisk, *P* > 0.05/2). (E) Fecundity of mites feeding on detached leaves of control (Kondara) and transgenic *Arabidopsis* lines expressing dsRNA-*TuVATPase* transgene over a 10-day period. Data were collected from 3 independent experimental runs and data were analysed using the Wilcoxon–Mann–Whitney test with Bonferroni correction (*, *P* < 0.05/2; ***, *P* < 0.001/2). (F) The expression of the endogenous *TuVATPase* gene relative to the expression of *RP49* reference gene. Data were represented as mean±SE and analysed using the Dunnett’s test (no asterisk, corrected *P* > 0.05).

*Arabidopsis* lines carrying a single (2–3) or multiple (2–1) transgenic loci were selected based on the segregation of the *bar* gene that confers resistance to the BASTA herbicide. This marker gene was co-integrated in *Arabidopsis* chromosomes with a transgene driving the constitutive expression of the hairpin dsRNA corresponding to the *TuVATPase-A* fragment. As expected, the dsRNA was transcribed in lines 2–1 and 2–3, but not in untransformed *Arabidopsis* control plants (Fig 4A). Since mite performance assays were done on detached leaves, we initially verified that the expression of the hairpin dsRNA-*TuVATPase-A* transgene was not affected by the leaf detachment from the plant in the course of the experiment (Fig 4B). Survivorship and transitions through developmental stages of the newly-hatched larvae were similar over a 10-day period irrespective of the leaf genotype on which they fed (Fig 4C and 4D). However, the fecundity was significantly lower in females fed on lines 2–1 and 2–3, respectively, compared to those that fed on Kondara control plants (Fig 4E), even though the *TuVATPase* transcripts levels were not significantly lower in spider mites fed on transgenic lines compared to the control (Fig 4F).

### Induction of RNAi in the leaf coating setup

**Fig 5:**
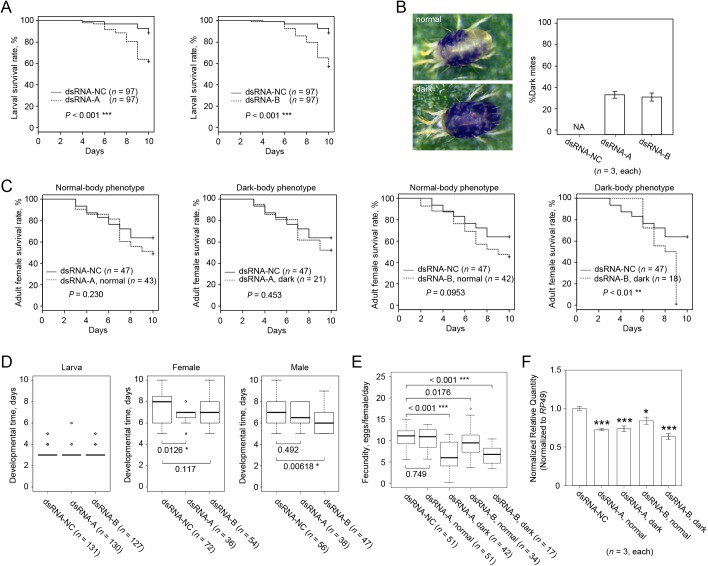
The effect of dsRNA-*TuVATPase* delivered by the leaf coating method. (A) Larval survivorship when feeding on bean leaf discs coated with the solution of dsRNA-*TuVATPase-*A, dsRNA-*TuVATPase-*B (dashed lines) or dsRNA-NC (solid line). Data were collected from 3 independent experimental runs. Survival curves were plotted using the Kaplan–Meier method and compared using the log-rank test with Bonferroni correction (***, *P* < 0.001/2). (B) Normal and dark-body female mites, and frequency of phenotypes observed after feeding on leaf discs coated with the solution of dsRNA-*TuVATPase-*A, dsRNA-*TuVATPase-*B or dsRNA-NC. The bar graph represents dark-body mite frequencies collected from 3 independent experimental runs. (C) Survivorship of adult female mites upon feeding on bean leaf discs coated with the solution of dsRNA-*TuVATPase-*A, dsRNA-*TuVATPase-*B (dashed lines) or dsRNA-*NC* (solid line) for normal and dark-body mites respectively. Data were collected from 3 independent experimental runs. Survival curves were plotted using the Kaplan–Meier method and compared using the log-rank test with Bonferroni correction (no asterisk, *P* > 0.05/4; **, *P* < 0.01/4). (D) Developmental times of the larval and adult female and male mites. Data were collected from 3 independent experimental runs and were compared using the Wilcoxon–Mann–Whitney test with Bonferroni correction (no asterisk, *P* > 0.05/2; *, *P* < 0.05/2; **, *P* < 0.01/2). (E) Normal and dark-body mite fecundity. Data were collected from 3 independent experimental runs and were compared using the Wilcoxon–Mann–Whitney test with Bonferroni correction (no asterisk, *P* > 0.05/4; ***, *P* < 0.01/4). (F) *TuVATPase* gene expression relative to the expression of *RP49* reference gene in normal and dark-body female mites. Data were represented as mean±SE and analysed using the Dunnett’s test (*, corrected *P* < 0.05; ***, corrected *P* < 0.001).

In leaf coating method, the solution of dsRNA is applied on the surface of the leaf disc, mimicking spray delivery common in agricultural practice. Although the foliar application of dsRNA can be effective against chewing herbivores [35], it was uncertain whether dsRNA coated on the leaf surface would be effective against mites that feed on plant mesophyll cells located within the leaf [36]. However, as shown in Fig 5A, larvae that fed on leaves coated with either dsRNA-*TuVATPase-*A or dsRNA-*TuVATPase-*B showed significantly lower survivorship relative to mites that fed on leaves coated with dsRNA-NC. In contrast to larvae fed on dsRNA-*TuVATPase* leaf discs, that had no visible phenotype, a portion of the adult mites exposed to the same dsRNAs applied to the leaf surface developed a dark-body color (Fig 5B). This phenotype was seen in 34% and 32% of mites treated with dsRNA-*TuVATPase-*A and dsRNA-*TuVATPase-*B, respectively, but was not observed in control population treated with dsRNA-NC. To test the association between the body color phenotype and RNAi effects, we followed mite performance separately on mites with normal (labeled as *normal* in Fig 5) and dark color (labeled as *dark* in Fig 5). Survivorship of adult mites treated with dsRNA-*TuVATPase-*A (regardless of the body-color phenotype) and dsRNA-NC was similar (Fig 5C). Likewise, survivorship of mites with normal body color treated with dsRNA-*TuVATPase-*B did not significantly differ from the control. However, the survivorship of dsRNA-*TuVATPase*-B treated mites displaying a dark-body phenotype was dramatically reduced (Fig 5C). Further, the effect of dsRNA-*TuVATPase* on mite fecundity was differential in mites with normal and dark-body phenotype. dsRNA-*TuVATPase* either did not affect (dsRNA-*TuVATPase-*A) or had a modest effect (dsRNA-*TuVATPase-*B) on fecundity of mites with normal body color. But, dark-colored mites, in treatments with either dsRNA-*TuVATPase-*A or -B fragments, had dramatically reduced fecundity (Fig 5E). The relative expression level of *TuVATPase* was reduced by dsRNA-*TuVATPase* relative to the control (Fig 5F). Even though mites with dark body color had more dramatic RNAi phenotypes relative to their counterparts with normal body color, expression levels of the endogenous *TuVATPase* correlated with the effect of RNAi only when mites were treated with dsRNA-*TuVATPase-*B. However, when mites were treated with dsRNA-*TuVATPase-*A, the reduction of the relative expression level in dark mites was comparable to that in mites with normal color.

### Induction of RNAi in soaked mites

In the methods described above, dsRNA intake into mites occurred through feeding. In parallel, we developed a direct dsRNA delivery method in which mites are soaked in the solution of dsRNA [23]. We showed that tracer dyes introduced into mites by soaking could be detected in the mite gut, and possibly in a wider domain, as a result of direct penetration [23].

**Fig 6:**
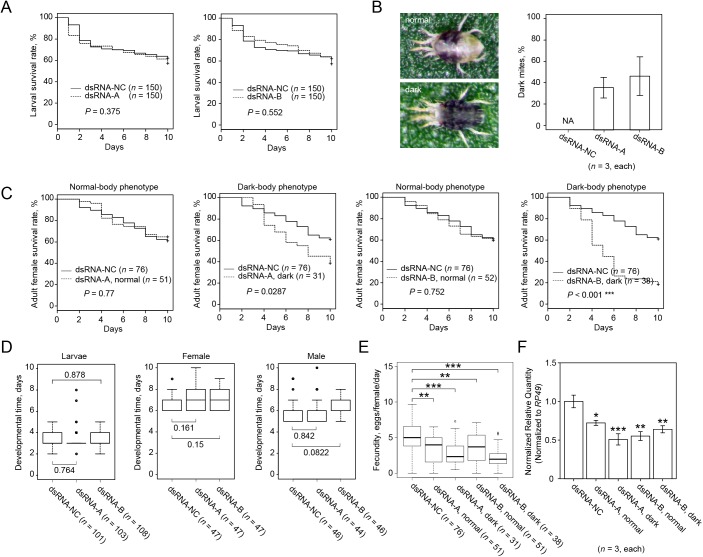
The effect of dsRNA-*TuVATPase* delivered by the soaking method. (A) Larval survivorship after soaking treatment in solution of dsRNA-*TuVATPase-*A, dsRNA-*TuVATPase-*B (dashed lines) or dsRNA-NC (solid line). Data were collected from 3 independent experimental runs. Survival curves were plotted using the Kaplan–Meier method and compared using the log-rank test with Bonferroni correction (no asterisk, *P* > 0.05/2). (B) Normal and dark-body female mites, and frequency of phenotypes observed after application of dsRNA-*TuVATPase-*A, dsRNA-*TuVATPase-*B or dsRNA-NC through soaking. The bar graph represents dark-body mite frequencies collected from 3 independent experimental runs. (C) Adult survivorship after soaking treatment in solution of dsRNA-*TuVATPase-*A, dsRNA-*TuVATPase-*B or dsRNA-NC separately for normal and dark-body mites. Survival curves were plotted using the Kaplan–Meier method and compared using the log-rank test with Bonferroni correction (no asterisk, *P* > 0.05/4; ***, *P* < 0.001/4). (D) Developmental times of the larval and adult female and male mites. Data were collected from 3 independent experimental runs and were compared using the Wilcoxon–Mann–Whitney test with Bonferroni correction (no asterisk, *P* > 0.05/2). (E) Normal and dark-body mite fecundity after soaking in solution of dsRNA-*TuVATPase-*A, dsRNA-*TuVATPase-*B or dsRNA-NC. Data were collected from 3 independent experimental runs and were compared using the Wilcoxon–Mann–Whitney test with Bonferroni correction (**, *P* < 0.01/4; ***, *P* < 0.001/4). (F) *TuVATPase* gene expression relative to the expression of *RP49* reference gene in normal and dark-body female mites. Data were represented as mean±SE and analysed using the Dunnett’s test relative to dsRNA-NC treatment (*, corrected *P* < 0.05; **, *P* < 0.01; ***, *P* < 0.001).

Larval survivorship of mites soaked in a dsRNA-*TuVATPase*-A or dsRNA-*TuVATPase*-B solution could not be distinguished from the control (Fig 6A). However, over 37% of the adults soaked in dsRNA-*TuVATPase*-A and 47% of adults soaked in dsRNA-*TuVATPase-B* developed the dark-body phenotype previously observed in mites that fed on bean leaf discs coated with a dsRNA-*TuVATPase* solution (Figs 5B and 6B). Adult mites with normal body color phenotype (*normal* in Fig 6) soaked in dsRNA-*TuVATPase*-A or dsRNA-*TuVATPase*-B had the same survivorship as mites soaked in the solution containing the control dsRNA-NC (Fig 6C). Although mites with the dark-body phenotype (*dark* in Fig 6) soaked with the dsRNA-*TuVATPase*-A fragment had a slightly lower survival than the control, the difference was not significant (Fig 6C). However, mites with the dark-body phenotype soaked with the dsRNA-*TuVATPase*-B fragment had a significantly lower survival than the control (Fig 6C). Developmental times of the larvae, females or males were unaffected by treatment (Fig 6D). The fecundity was significantly affected by mite soaking in the solution containing the dsRNA-*TuVATPase* (Fig 6E) and was more prominently reduced in mites with dark than with normal body color. In addition, the endogenous *TuVATPase* transcripts were significantly reduced in the dsRNA-*TuVATPase* treated mites (Fig 6F). Like with mites that received dsRNA through leaf coating, the severity of RNAi phenotypes correlated with the residual levels of the endogenous *TuVATPase* only upon treatment with one of the dsRNA fragments, the dsRNA-*TuVATPase-*A (Fig 6F).

### Differential RNAi response in *T*. *urticae* inbred lines

Application of dsRNA-*TuVATPase* by leaf coating and soaking resulted in the phenotypic change of mite body color that correlated with the reduced mite fitness (survivorship, fecundity). Given that changes in body color occurred only in mites treated with dsRNA-*TuVATPase* and not when mites were soaked in the control dsRNA-NC fragment, the observed phenotype is not a general response to dsRNA, but a specific response to the RNA interference with *TuVATPase* expression.

These assays involved mite larvae and adults that were tightly synchronized in their development (within a 3-hour interval) originating from the London mite population whose genetic variability is narrow. Nevertheless, the strong RNAi effects (characterized by dark-body color, and significant increase of mortality and reduced fecundity) were observed only in a portion of the treated mites. When dsRNA-*TuVATPase* was coated on the bean leaf, around 30% of adults showed the strong RNAi effects, while mite soaking in the solution of dsRNA-*TuVATPase* increased the proportion of the affected adults to 50%. Such partial penetrance of the RNAi-induced phenotypes may be explained in 2 ways: a) it is an intrinsic property of the delivery methods; or b) genetic factors control the RNAi response and are variable in the London mite population.

**Fig 7:**
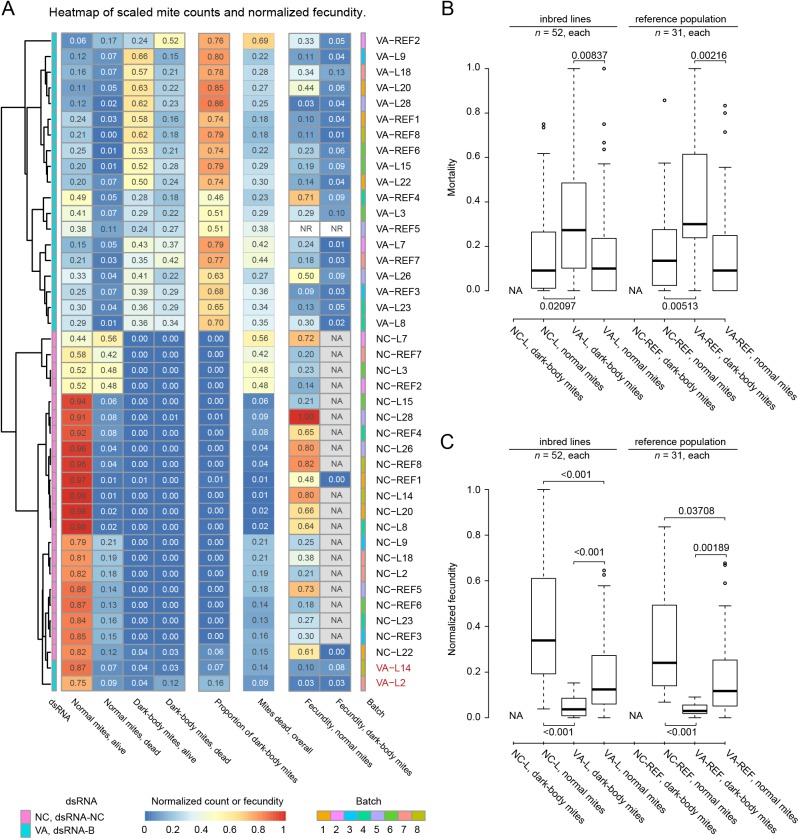
RNAi response in soaked *T*. *urticae* inbred lines. (A) Experimental combinations of 14 mite groups (13 inbred lines and the reference population) with 2 dsRNAs (dsRNA-*TuVATPase-*B (label VA) and dsRNA-NC control (label NC)) were classified using hierarchical average clustering and Euclidean distance based on normalized survivorship (dead/alive counts) and body color (proportion of dark mites). The mite/dsRNA combinations were tested in 8 experimental batches (indicated by colors at the right side of the panel) and analyzed together. Each run included a sample of the London reference population (label REF) and 1 or 2 inbred line samples (label L). Normalized fecundity over 3 days and total proportion of dark mites are shown in the same order as dead/alive counts but were not taken into account for clustering. In most dsRNA-NC control treatments dark-body mites were not present and their fecundity could not be measured (not applicable, NA). Fecundity for VA-REF5 samples was not recorded (NR) due to a technical issue. Data were collected from 4 independent experimental runs in 8 batches. All measurements are expressed as ratios relative to the total number of tested mites and represented acccordingly to the heatmap color code (bottom). (B) Normal and dark-body mite mortality (proportion of dead mites) in inbred lines (label L) and the reference population (label REF) treated with 2 dsRNAs (dsRNA-*TuVATPase-*B (label VA) and dsRNA-NC control (label NC)). (C) Normal and dark-body mite fecundity in inbred lines and the reference population treated with dsRNA-*TuVATPase-*B (label VA) and dsRNA-NC control (label NC). Data from 8 experimental batches was analyzed together and separated at the level of mite line type (REF and L), treatment (NC and VA), and phenotype (normal and dark-body) classes. As in most dsRNA-NC control treatments dark-body mites were not present their mortality and fecundity could not be measured (not applicable, NA). All *P*-values shown are corrected for multiple comparisons.

To test the latter hypothesis, we analyzed the RNAi response of thirteen independent inbred (over 6 generations) lines derived from the London population. For each inbred line, newly-emerged adult female mites were soaked in a solution containing either the dsRNA-*TuVATPase*-B or dsRNA-NC fragment. Mites with normal or dark-body phenotypes were then counted and the fitness (mortality and fecundity) of both classes was measured separately. Genotype/dsRNA combinations were clustered according to survivorship and body color (Fig 7).

The highest level clusters clearly partitioned treatments with dsRNA-*TuVATPase*-B (top) and the control dsRNA-NC (bottom) (Fig 7A). Within these 2 major groups, mite genotypes (London population and inbred lines) were intermixed (Fig 7A) and their performance values were similar (Fig 7B and 7C), indicating that inbred lines reconstitute genetic variability existing in the London population.

The RNAi response triggered by dsRNA-*TuVATPase*-B was characterized by dark mites that invariably had higher mortality (Fig 7B) and lower fecundity (Fig 7C) relative to control or normal mites within the same population, further strengthening the link between the silencing of the *TuVATPase* gene with the color body phenotype and performance parameters. However, mites with a normal body color (*normal* in Fig 7) treated with dsRNA-*TuVATPase*-B also displayed an effect of the RNAi, albeit at reduced level. Their mortality is indistinguishable from the control population (Fig 7B), however, their fecundity is significantly reduced (Fig 7C). Thus, mite populations treated with the dsRNA-*TuVATPase*-B partition in 2 phenotypic classes (dark and normal body color) that differ in the quantitative severity of the RNAi effects.

None of the dsRNA-*TuVATPase*-B treated populations displayed 100% of dark mites, indicating that the high RNAi-efficiency is not completely penetrant and that the variability is intrinsic to the treatment. Two inbred lines (L2 and L14) were peculiar outliers that clustered with the control dsRNA-NC treatments, yielding no or very few dark mites. However, despite a normal color phenotype, these mite lines had similar mortality and fecundity as other inbred lines and London population, indicating that they respond to RNAi but seemingly lack the ability to display a dark-body phenotype.

## Discussion

Even though *T*. *urticae* reverse genetics protocols describing the maternal injection and oral delivery of dsRNA have been published [15,18,37], they are not suitable for high throughput screens. In this study, we tested 4 methods based on artificial diet, transgenic plants, leaf coating or soaking to deliver dsRNA into spider mites. The efficiency of these methods was compared to the floating leaf disc assay, with the same dsRNA-*TuVATPase* fragment and concentration previously reported by Kwon *et al*. [18].

**Fig 8:**
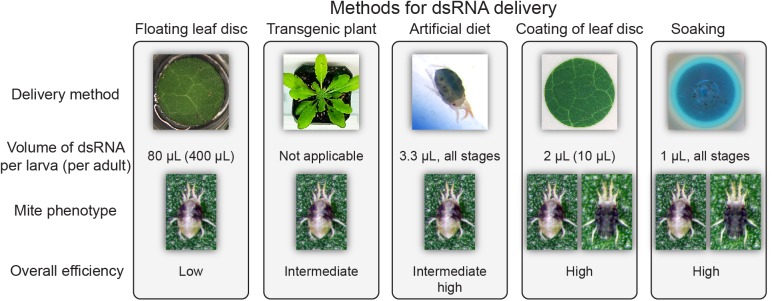
dsRNA delivery methods for RNAi-based gene silencing in *T*. *urticae*. Graphical summary of required dsRNA amount, severity of phenotype, and overall efficiency for the 5 dsRNA delivery methods compared in this study.

Several mite fitness parameters (larval and adult survivorship, larval and adult developmental timing and fecundity) and the endogenous targeted transcript level were measured to determine RNAi efficacy. As our aim was to assess relative performance of delivery methods and RNAi efficiency, we utilized conservative methods of statistical analysis. In our hands, the 4 alternative methods yielded higher silencing and/or stronger phenotypes than the floating leaf disc assay (Figs 2–6 and 8). The superior efficiency of these methods is reflected in: a) the lower amount of dsRNA applied (80 **μ**L per larva of dsRNA solution in the floating leaf disc assay *vs*. 1 to 4 **μ**L in the others), and b) the severity of the RNAi effects on mite fitness and/or endogenous target silencing. dsRNA-*TuVATPase* applied in the floating leaf disc assay had no significant effects on any of the recorded fitness parameters (Fig 2). Induction of RNAi through the expression of the dsRNA-generating transgene also had limited effect on mites: of all parameters tested, only fecundity was significantly reduced by mite feeding on transgenic relative to the control plants (Fig 4). Lower larval feeding intensity on *Arabidopsis* and potentially lower concentration of dsRNA present in transgenic plant tissues can explain the reduced effect of transgene-expressed dsRNA. Overall, the fecundity was the most sensitive parameter indicative of RNAi that was significantly reduced upon dsRNA delivery through transgenic plants, leaf coating or soaking (Figs 4, 5 and 6; note that this parameter could not have been tested for delivery of dsRNA via artificial diet, as female mites are not fecund under this rearing method).

Our comparative analysis identified soaking and leaf coating as 2 most effective ways to induce RNAi in *T*. *urticae* and application of dsRNA-*TuVATPase* with either method resulted in reduction of mite survivorship, fecundity and target gene expression (Figs 5 and 6). Similar dsRNA-*VATPase* silencing phenotypes have been described across multiple experimental systems, e.g. whiteflies, western corn rootworms, western flower thrips, Colorado potato beetles, fruit flies, flour beetles, pea aphids, and tobacco hornworms [12,20–22,38,39]. They confirm the essential role of the VATPase pump in proton transport across cellular membranes of eukaryotic organisms [19]. However, in addition to these phenotypes, and unique to this study, the silencing of *TuVATPase* resulted in a dark-body phenotype (Figs 5 and 6). The effect of dsRNA-*TuVATPase* on body pigmentation may reflect distinctive features of mite physiology. It may also be a more general phenomenon that is only now observed in mites because they have semitransparent integument. In our study, the dark-body phenotype tightly correlated with dsRNA-*VATPase* treatment and high RNAi effectiveness (Figs 5–7). However, it could also be observed in aging mites in untreated mite populations (note that adult mites used in our study were newly molted adult females). Thus, the dark-body phenotype is not specific to the disruption of the *TuVATPase* gene function, but may reflect mite body color change that associates with stress that can be induced by multiple independent ways.

Only a portion of mites treated with the dsRNA-*VATPase-*B fragment developed a dark body phenotype (Figs 5–7). The incomplete phenotype penetrance is likely due to the differential ability to: a) deliver dsRNA into a portion of the treated mites, or b) to silence the target gene. We previously used tracer dyes to track the delivery of small molecules into the mite body and have observed differential dye accumulation in the treated mites [15,23]. In addition, we measured that siRNA designed to silence *Tu-Dll* induced a reduction of gene expression and caused the loss-of-function phenotype in ~60% of mite embryos that received the siRNA [15]. The partial RNAi efficiency was again observed in this study despite the homogeneity of the mite genotype (inbred lines) and the tight synchronization of mite development in the experimental population (Figs 5–7). The instability of dsRNA due to the presence of nucleases, variable expression levels, or mutations in genes encoding the RNAi machinery have been shown to affect RNAi in several experimental systems [40–42]. In addition, the sequence variability of gene regions targeted for interference and differential physiological backgrounds have been postulated as factors that affect RNAi efficiency [43]. Further studies will be required to understand the source of partial and variable RNAi efficiency in spider mites.

Our analysis further highlighted that genetic component(s) can modulate RNAi-induced responses in mites. Two out of 13 inbred lines soaked in the solution containing dsRNA-*VATPase-*B did not develop the dark-body phenotype. However, the fecundity of these mites was significantly reduced relative to the dsRNA-NC treated controls (Fig 7), indicating effective RNAi despite the inability of these mites to develop a dark-body phenotype. The dark-body phenotype in dsRNA-*VATPase-*B responsive mite populations may be used as an easily assessable trait to compare factors such as RNA chemical modifications, transfection reagents and carriers when optimizing dsRNA penetration into mites.

The analysis of the *T*. *urticae* genome sequence identified many genes that either belong to expanded known gene families or that are “orphan” genes, with no obvious orthologs, often restricted to certain taxons [5]. Efficient reverse genetics platforms are urgently needed to study the biological and evolutionary role of these uncharacterized sequences. In this perspective, the 4 delivery methods presented here could be adapted for the application of dsRNA as a reverse-genetics tool for spider mite that will be an important asset for both the fundamental and applied sciences.

*T*. *urticae* is a pest with a staggering host range and one of the pest arthropod species that is most resistant to chemical pesticides [44,45]. It is thus a prime model to study the evolution of host range, plant-herbivore interactions and mechanisms of xenobiotic resistance. RNAi itself, applied in fields or greenhouses, could prove to be a valuable and sustainable biotechnological approach for pest control [18,35,37]. For this approach, future investigations on design of dsRNA consisting of pest-lethal sequences that are highly specific to the pest species and use of a carrier for increasing stability of dsRNA will be needed.
